# 23335 Using AMPAC Score and Age to Identify Potential Over-utilization of PT Consults by Hospitalists

**DOI:** 10.1017/cts.2021.540

**Published:** 2021-03-30

**Authors:** Maylyn Martinez, Manoor Baig, Matthew Cerasale, Claire Dugan, Meghan Sweis, Mala Robinson, Vineet Arora

**Affiliations:** 1 University of Chicago; 2 University of Illinois, Chicago

## Abstract

ABSTRACT IMPACT: This work underscores the importance of judicious utilization of inpatient therapy services as a means to keep patients MORE independent and prevent readmissions OBJECTIVES/GOALS: We aimed to assess the potential over-utilization of physical therapy consults on a hospital medicine service using validated Activity Measure Post Acute Care (AM-PAC) score cutoffs. METHODS/STUDY POPULATION: We conducted a chart review of all patients admitted to the uncovered hospital medicine services at a large academic hospital for one year. For patients who had a PT consult at any time during their admission we obtained age, admission AMPAC score, and discharge destination. PT consults were considered ‘potential overutilization’ for AMPAC scores >/=19 based on previous studies validating this cutoff for predicting discharge to home. Descriptive statistics were used to summarize % of patients < 65 years old vs. >/=65 years and % of patients discharged to home vs. post-acute care. Multivariable logistic regression was used to examine independent associations between age group, AMPAC group, and an interaction term (age group x AMPAC group) with odds of being discharged home. RESULTS/ANTICIPATED RESULTS: Of 6,634 patients admitted during the year, 58% (n=3582) had a PT consult. Mean age was 66.3 +/-15.4 and mean AMPAC was 18.3 +/- 5.3. Seventy percent were discharged home (N=2497). Using AMPAC of >/= 19, 55% of consults were ‘potential overutilization’. Patients <65 with AMPAC>19 represented 31% of PT consults. AMPAC>19 had increased odds of discharge home (OR 3.58 [95% CI=2.17 -5.91]; P<0.001) as did age <45 years (OR 1.81 [95% CI=1.09-3.00]; P=0.02). A significant interaction existed between all ages and AMPAC>/=19 (For age<45 OR 2.85 for discharge home [95% CI=1.37 -4.30] P=0.002; For age 46-64 OR 2.43 for discharge home [95% CI=1.37-4.34] P=0.002). Combining age with AMPAC>/=19 had additional predictive value for discharge home (Pr=89% [95% CI 81%-97%] using age<45 vs. (Pr=83% [95% CI 77%-90%]) using age<45 alone. DISCUSSION/SIGNIFICANCE OF FINDINGS: Many PT consults may represent potential over-utilization. Avoiding these could save hundreds of PT hours per year by conservative estimate. Combining age with AMPAC scores can help predict who may not require a PT consult. Reallocating PT resources to the patients who do require it can help prevent functional decline and readmissions.

